# Supplementary material for: Four New Depsides Isolated from Salvia miltiorrhiza and Their Significant Nerve-Protective Activities
Source: Molecules. 2018 Dec 11;23(12):3274. doi: 10.3390/molecules23123274 (PMC6320994; doi:10.3390/molecules23123274)

SUPPLEMENTARY DATA

Four new depsides isolated from *Salvia miltiorrhiza* and their significant nerve-protective activities

Qinghao Jin[a,1], Xinyi Hu[a,b,1], Yanping Deng[a], Jinjun Hou[a], Min Lei[a], Hongjian Ji[a,c], Jing Zhou[a], Hua Qu[a], Wanying Wu[a, *], Dean Guo[a **]

[1] Shanghai Institute of Materia Medica, Chinese Academy of Sciences, Haike Road #501, Shanghai 201203, China.

[2] Institute of Bioengineering, Zhejiang University of Technology, Hangzhou, Zhejiang Province 310014, PR China

[*] Correspondence: wanyingwu@simm.ac.cn (W.-Y.W.); daguo@simm.ac.cn (D.-A.G.);

[†] The authors contribute equally to this work.

Supporting Information Contents:

S1. The ^{1}H NMR spectrum of **1** (CD_3OD, 500 MHz)

S2. The ^{13}C NMR spectrum of **1** (CD_3OD, 125 MHz)

S3. The HSQC spectrum of **1** (CD_3OD)

S4. The HMBC spectrum of **1** (CD_3OD)

S5. The HR-ESI-MS spectrum of **1**

S6. The CD spectrum of **1**

S7. The ^{1}H NMR spectrum of **2** (DMSO-d_6, 500 MHz)

S8. The ^{13}C NMR spectrum of **2** (DMSO-d_6, 125 MHz)

S9. The HSQC spectrum of **2** (DMSO-d_6)

S10. The HMBC spectrum of **2** (DMSO-d_6)

S11. The HR-ESI-MS spectrum of **2**

S12. The ^{1}H NMR spectrum of **3** (DMSO-d_6, 500 MHz)

S13. The ^{13}C NMR spectrum of **3** (DMSO-d_6, 125 MHz)

S14. The HSQC spectrum of **3** (DMSO-d_6)

S15. The HMBC spectrum of **3** (DMSO-d_6)

S16. The HR-ESI-MS spectrum of **3**

S17. The ^{1}H NMR spectrum of **4** (DMSO-d_6, 500 MHz)

S18. The ^{13}C NMR spectrum of **4** (DMSO-d_6, 125 MHz)

S19. The HSQC spectrum of **4** (DMSO-d_6)

S20. The HMBC spectrum of **4** (DMSO-d_6)

S21. The HR-ESI-MS spectrum of **4**

S1. The ^{1}H NMR spectrum of **1** (CD_3OD)

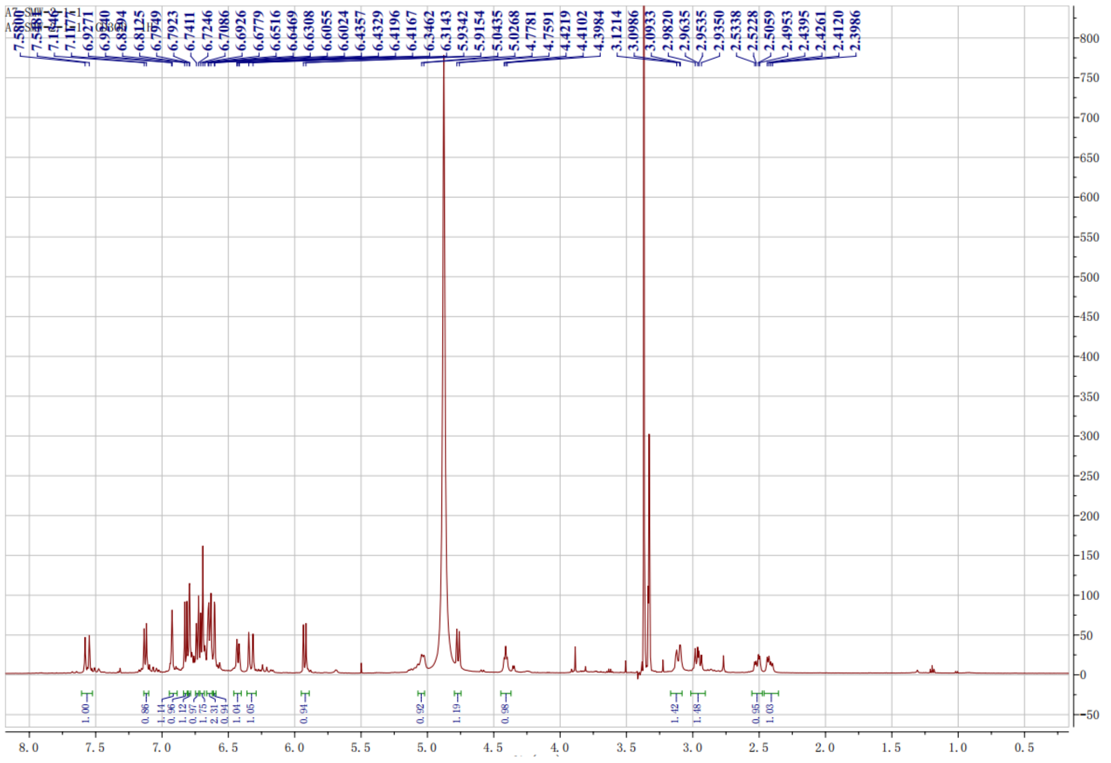

S2. The ^{13}C NMR spectrum of **1** (CD_3OD)

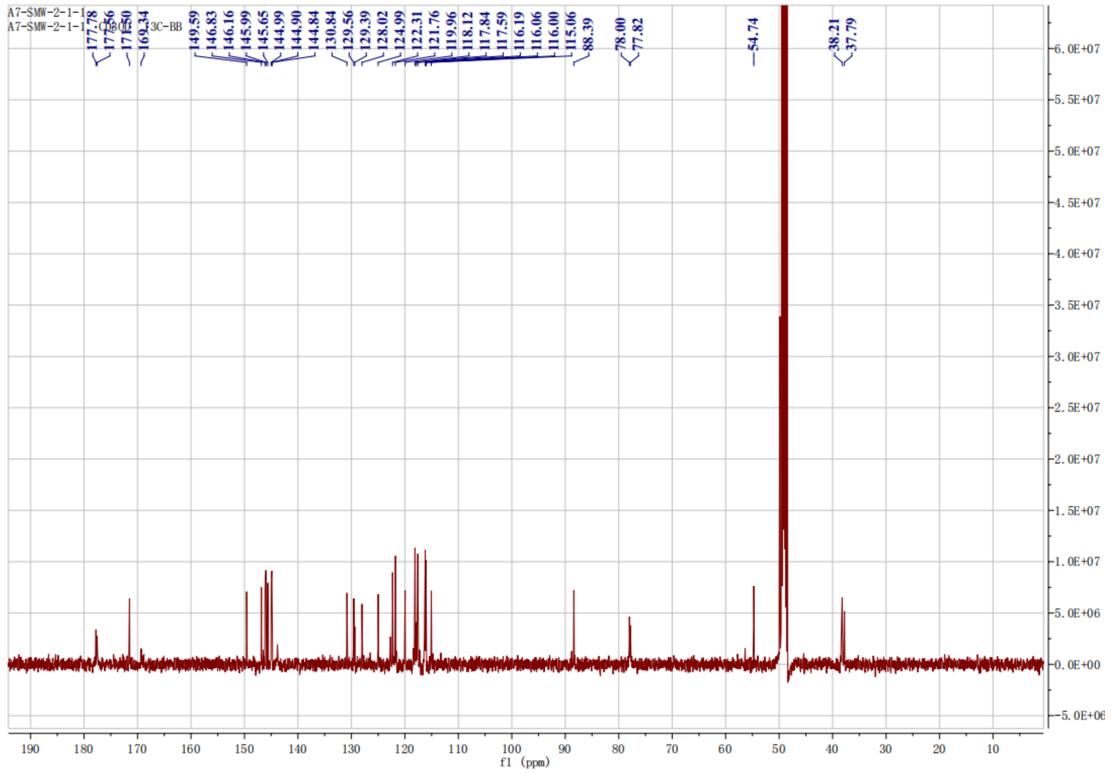

S3. The HSQC spectrum of **1** (CD_3OD)

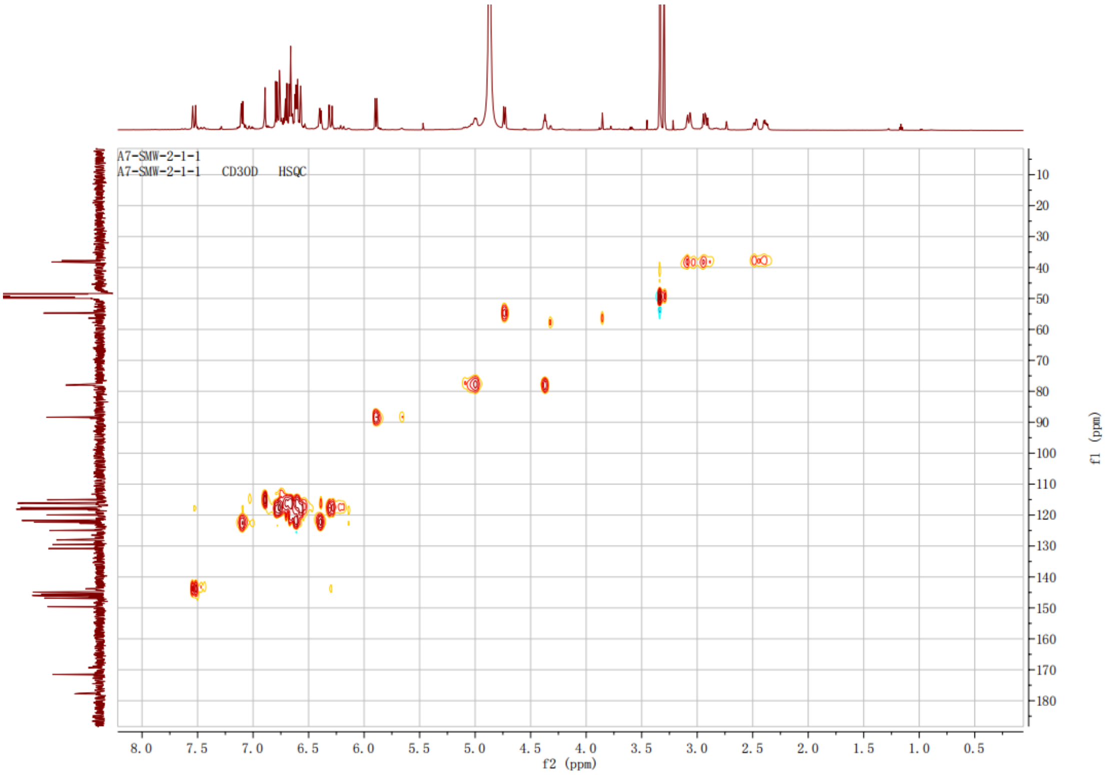

S4. The HMBC spectrum of **1** (CD_3OD)

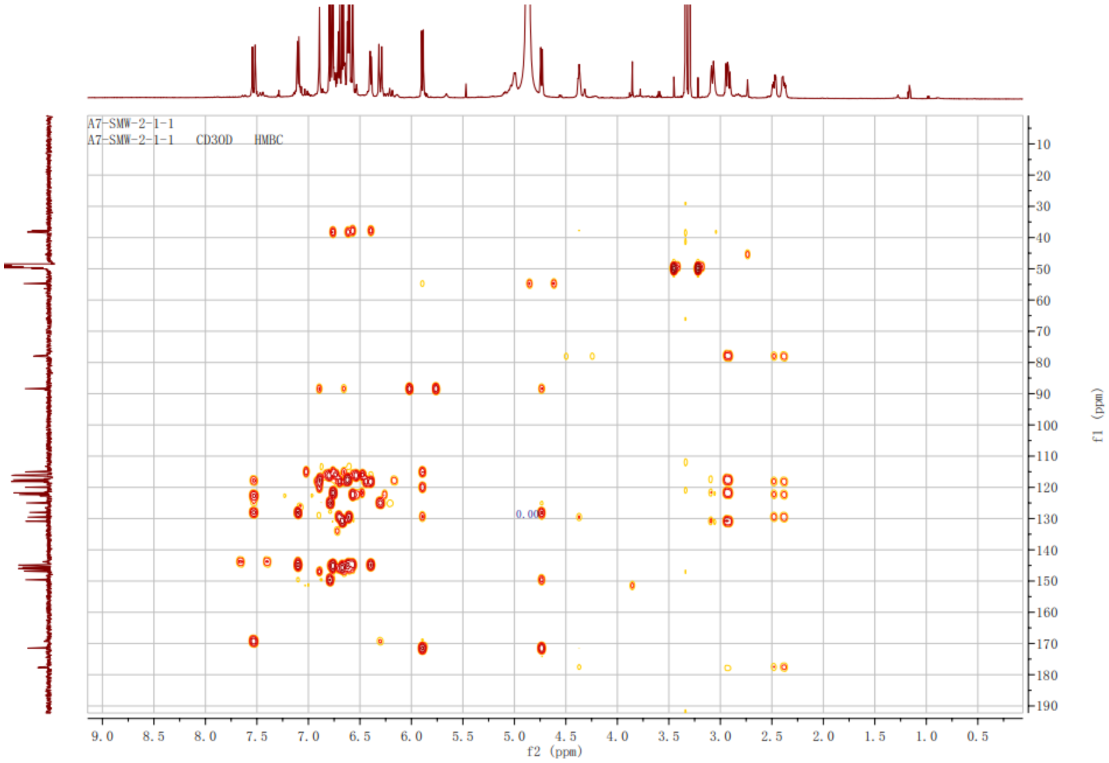

S5. The HR-ESI-MS spectrum of **1**

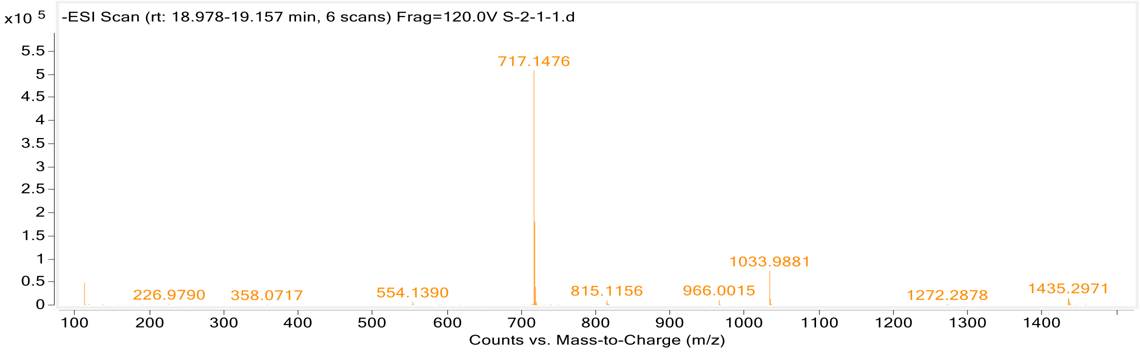

S6. The CD spectrum of **1**

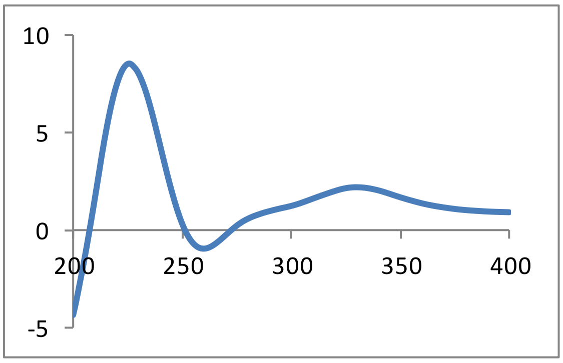

S7. The ¹H NMR spectrum of **2** (DMSO-d_6, 500 MHz)

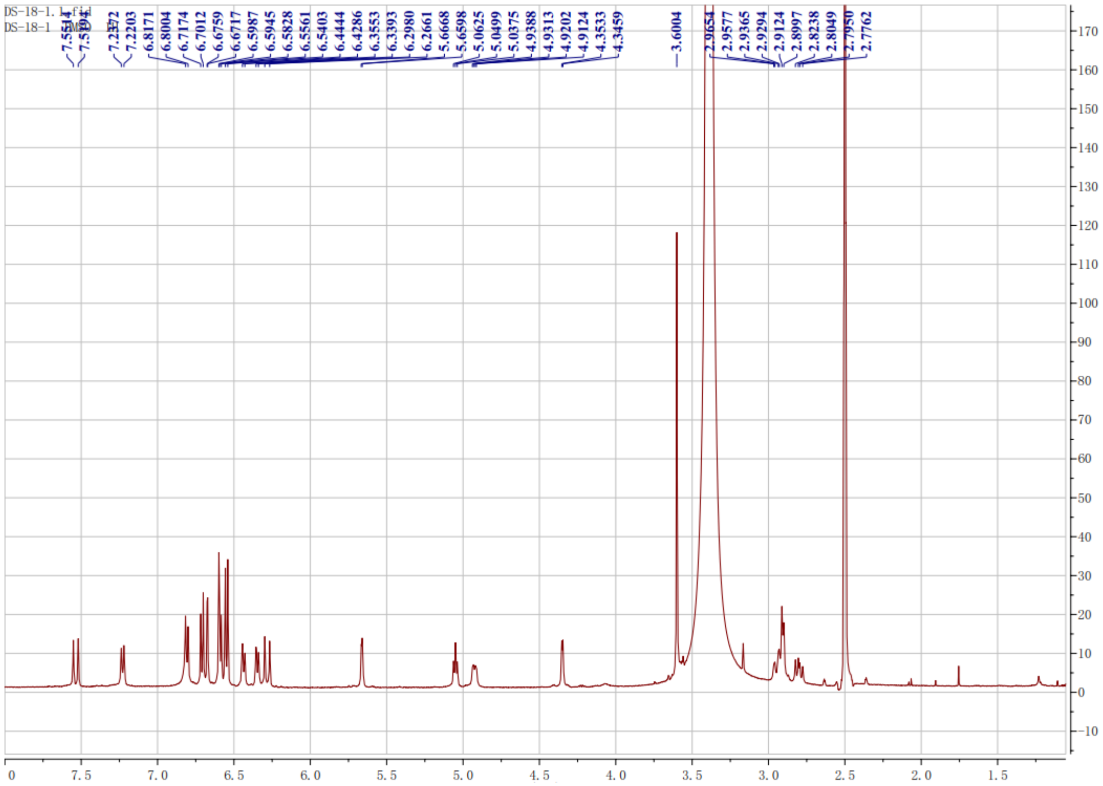

S8. The ^{13}C NMR spectrum of **2** (DMSO-d_6, 125 MHz)

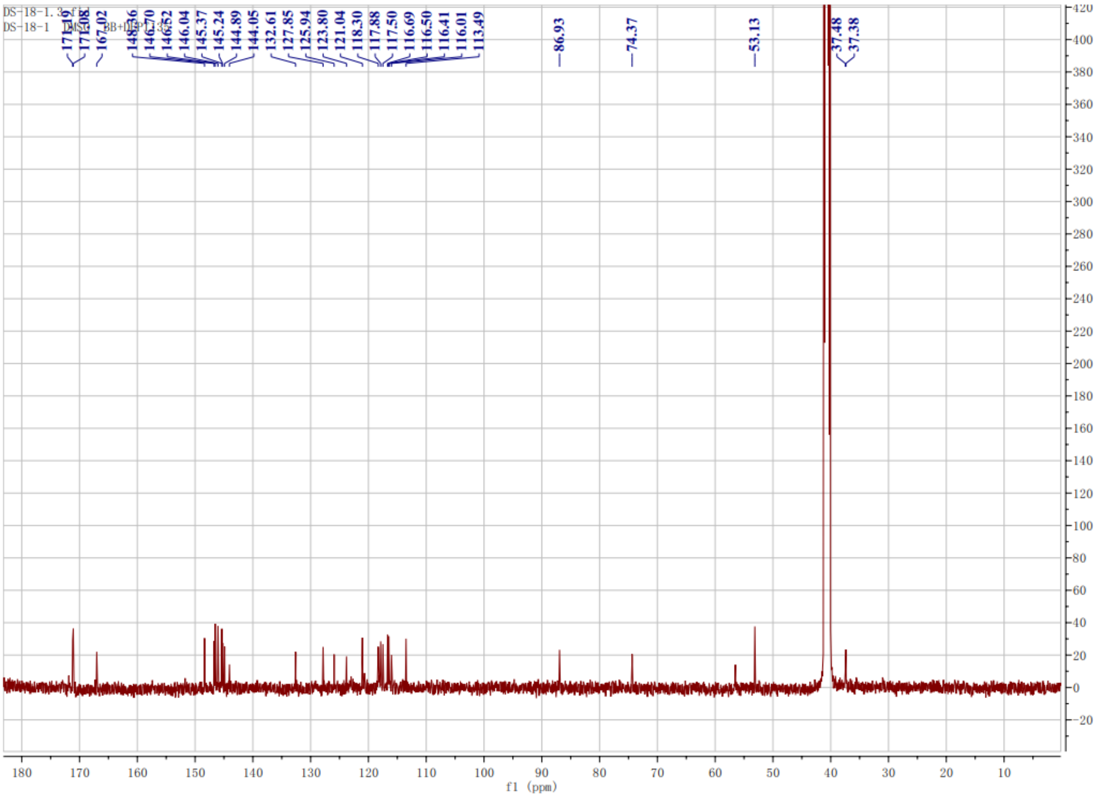

S9. The HSQC spectrum of **2** (DMSO-d_6)

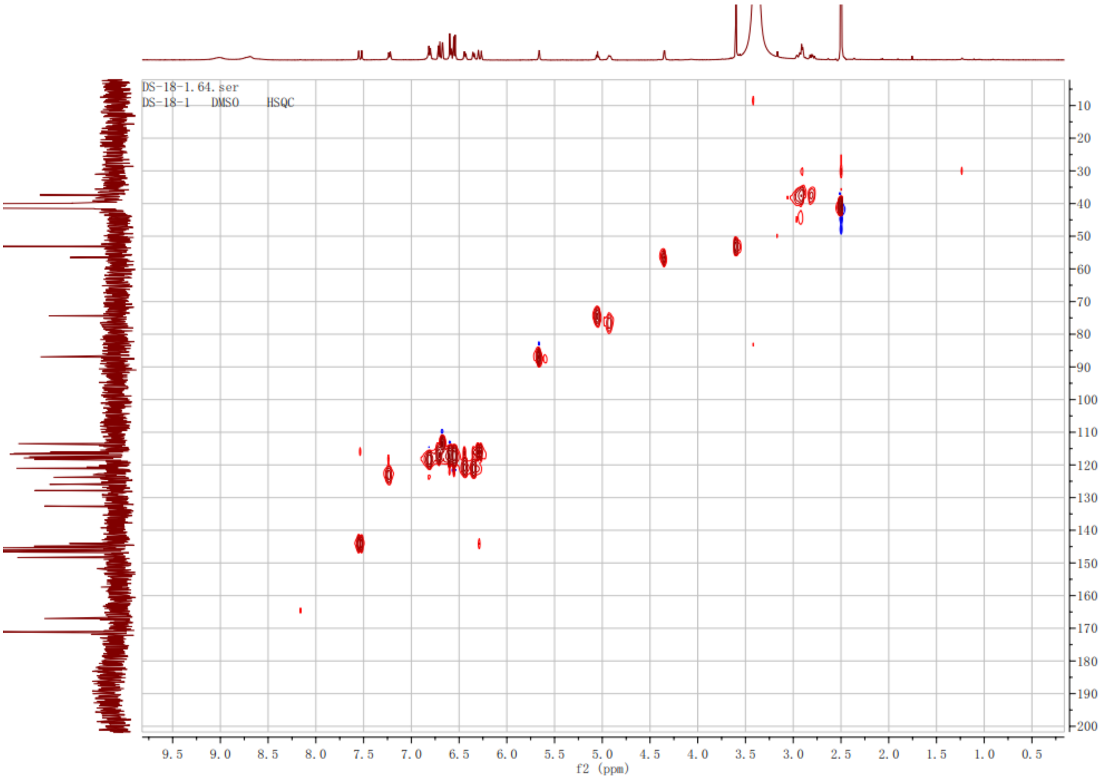

S10. The HMBC spectrum of **2** (DMSO-d_6)

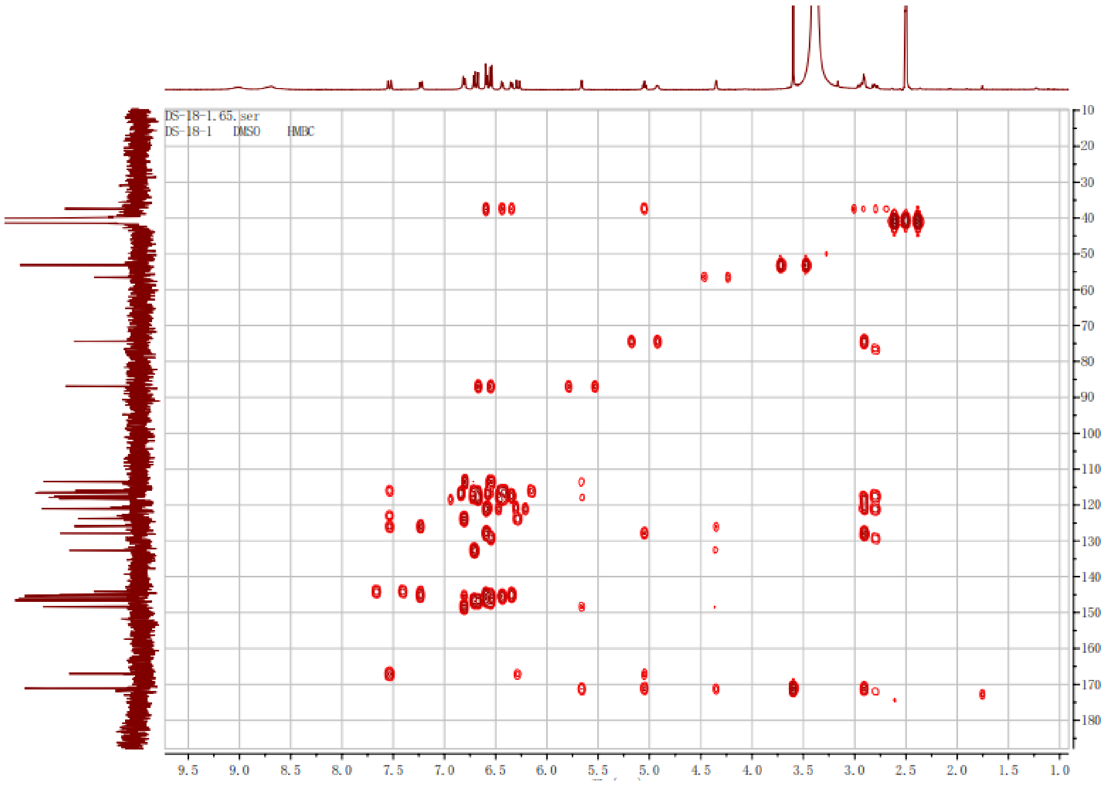

S11. The HR-ESI-MS spectrum of **2**

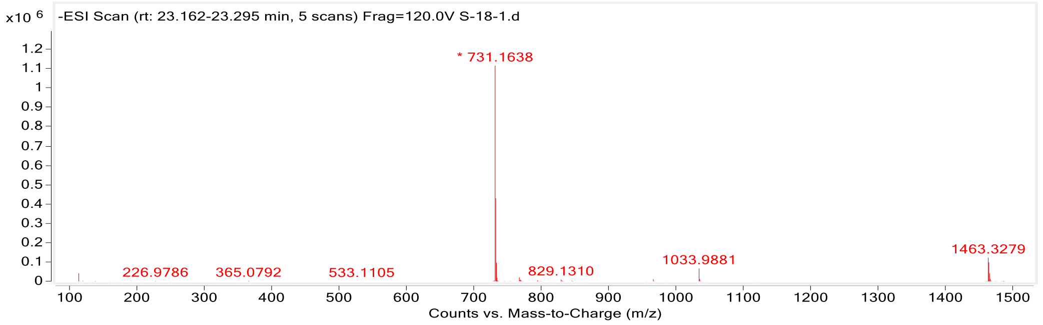

S12. The ^{1}H NMR spectrum of **3** (DMSO-d_6, 500 MHz)

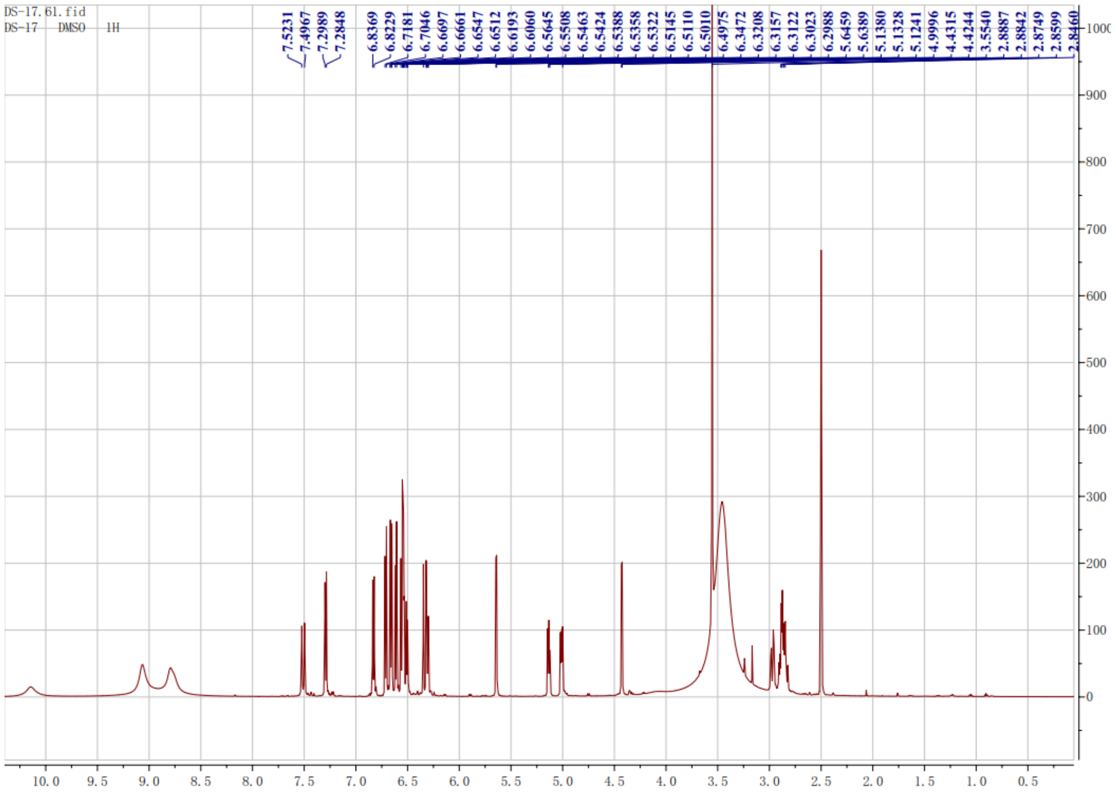

S13. The ^{13}C NMR spectrum of **3** (DMSO-d_6, 125 MHz)

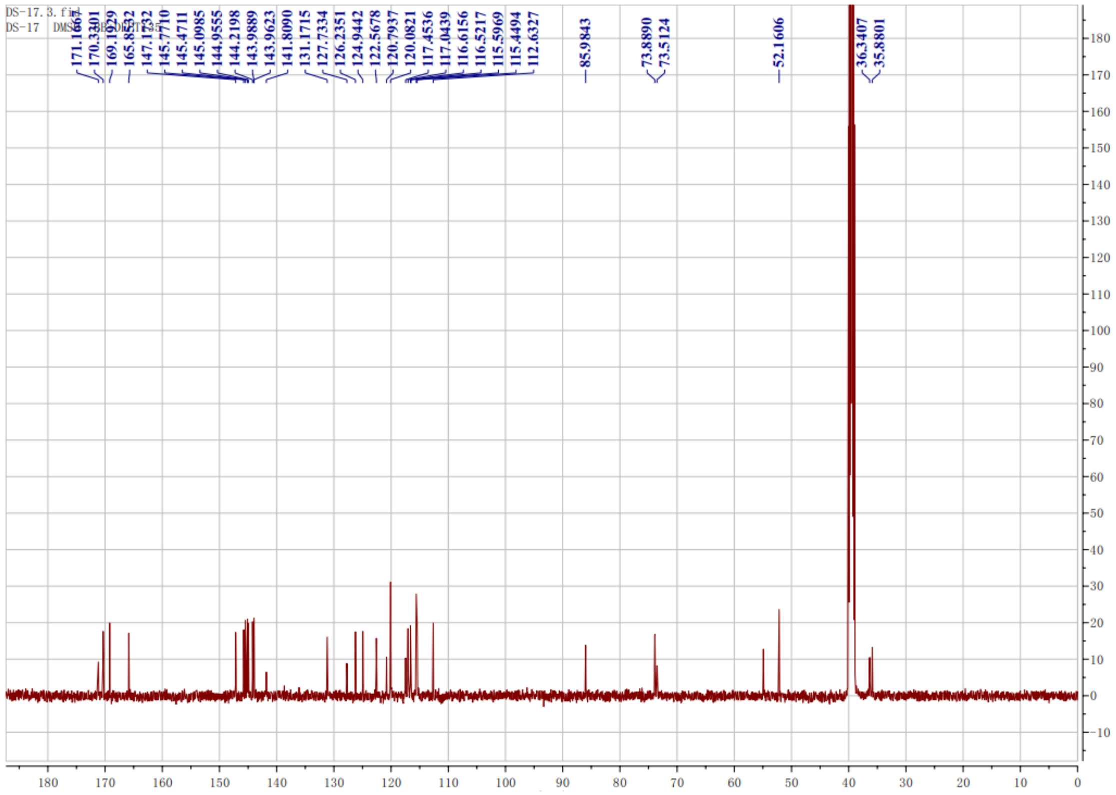

S14. The HSQC spectrum of **3** (DMSO-d_6)

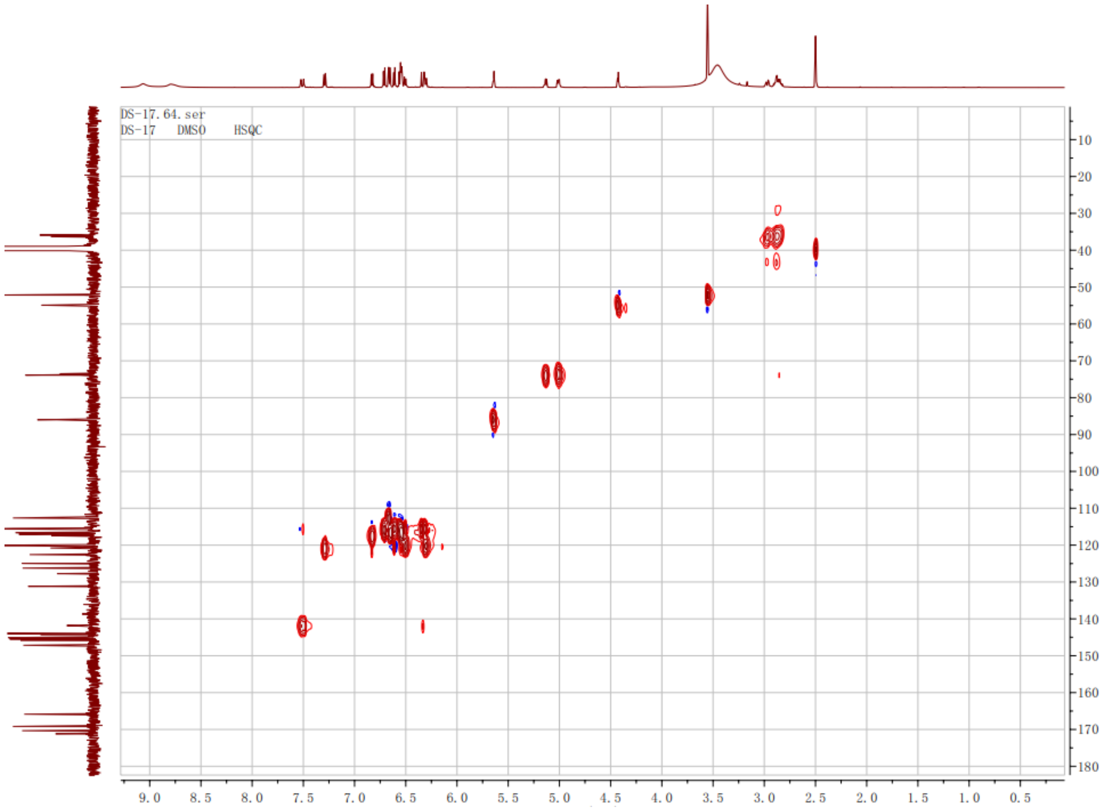

S15. The HMBC spectrum of **3** (DMSO-d_6)

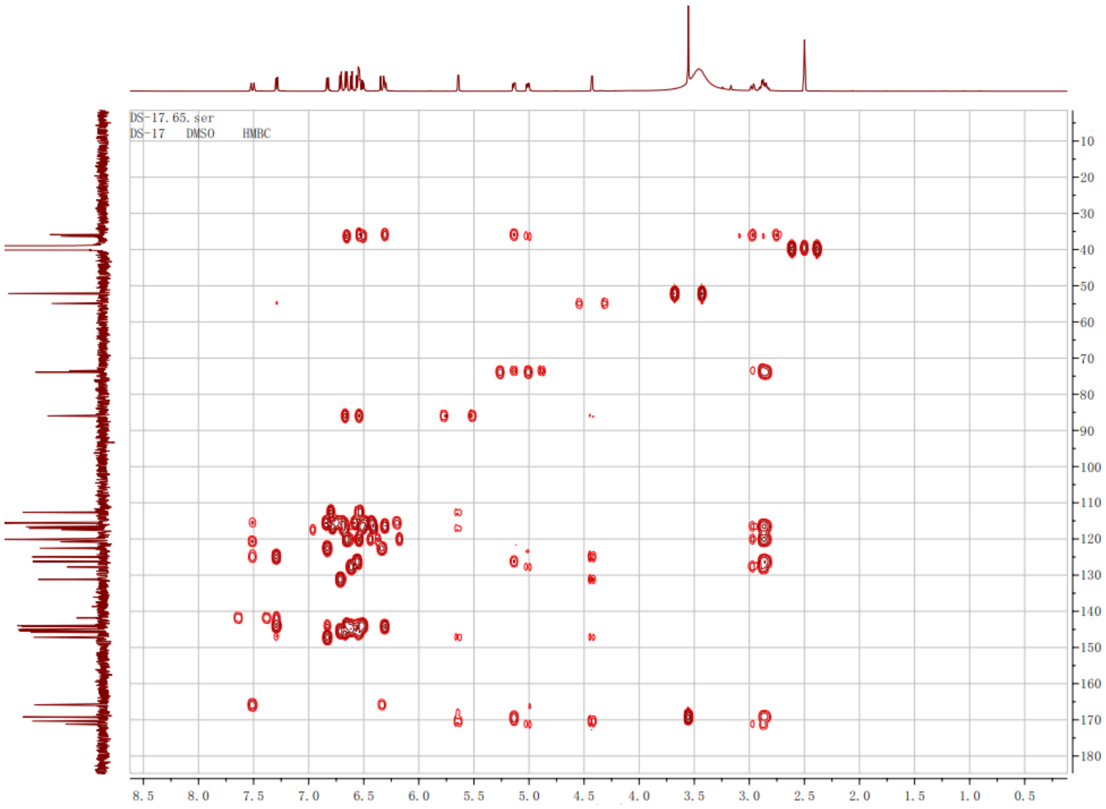

S16. The HR-ESI-MS spectrum of **3**

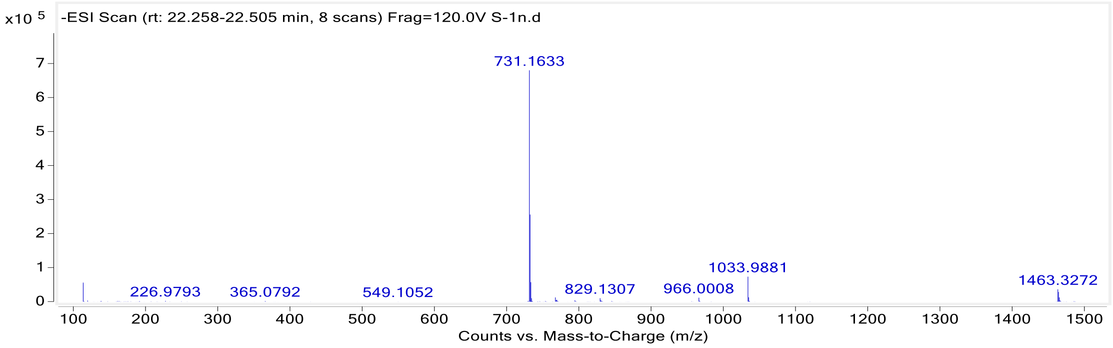

S17. The ^{1}H NMR spectrum of **4** (DMSO-d_6, 500 MHz)

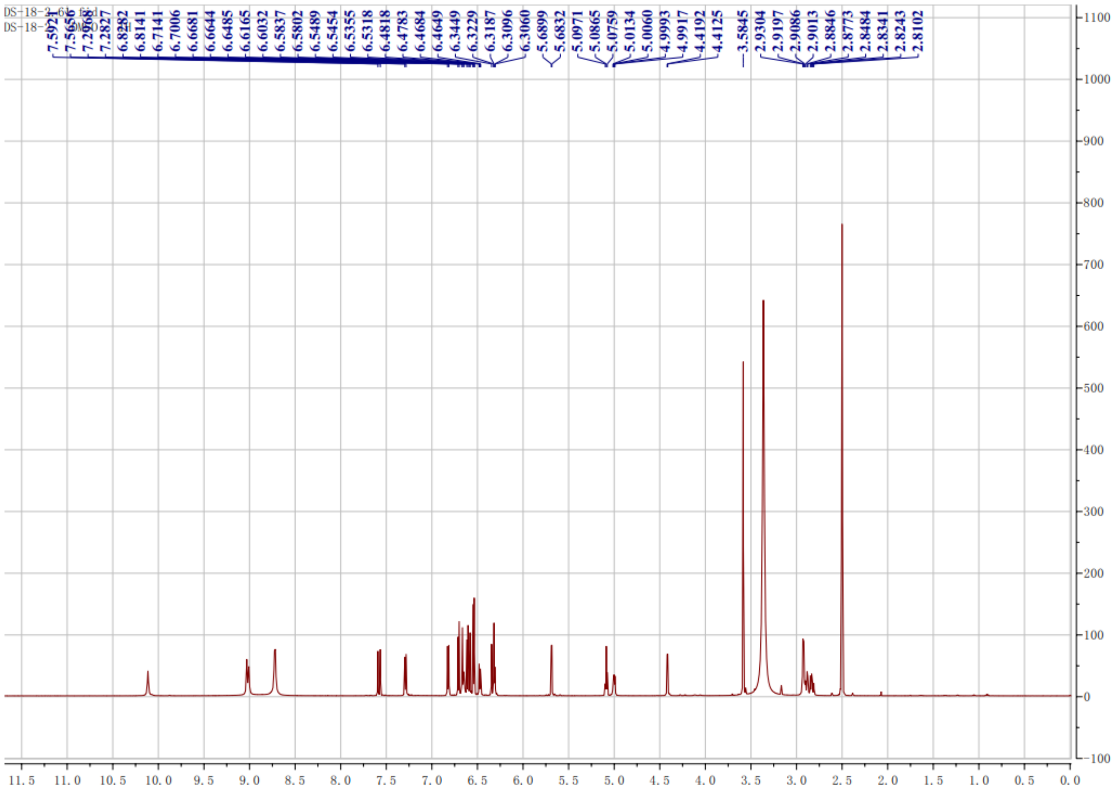

S18. The ^{13}C NMR spectrum of **4** (DMSO-d_6, 125 MHz)

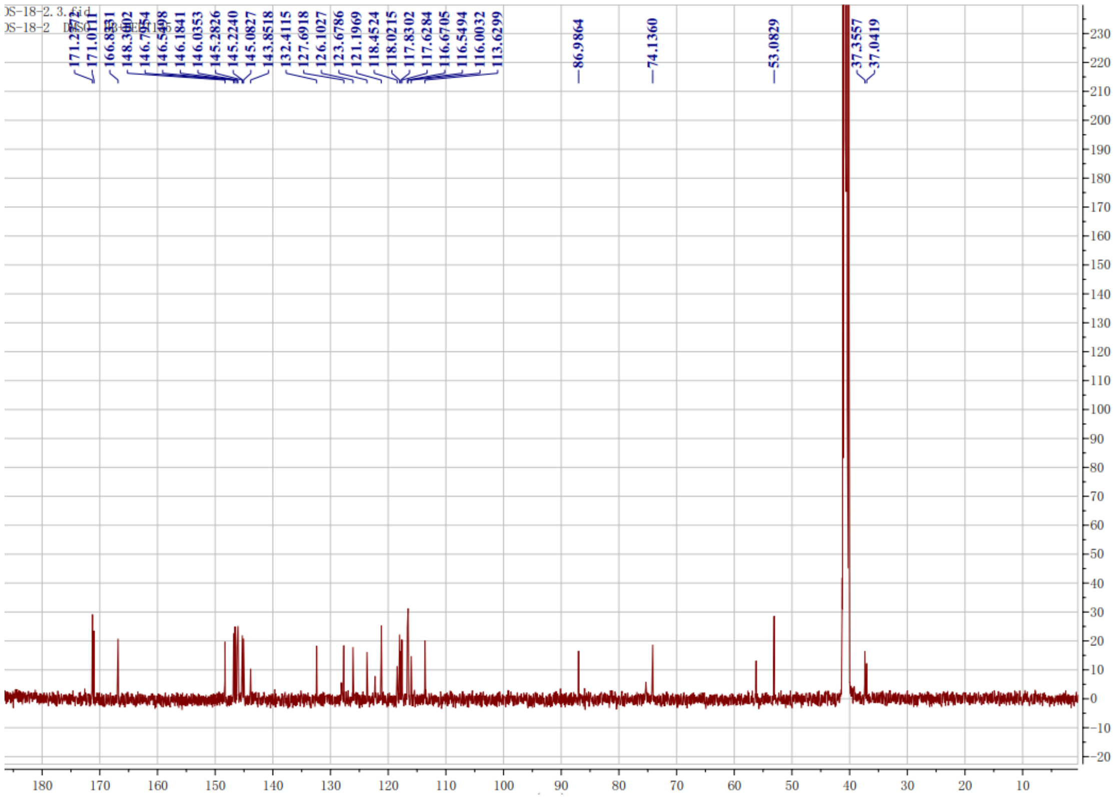

S19. The HSQC spectrum of **4** (DMSO-d_6)

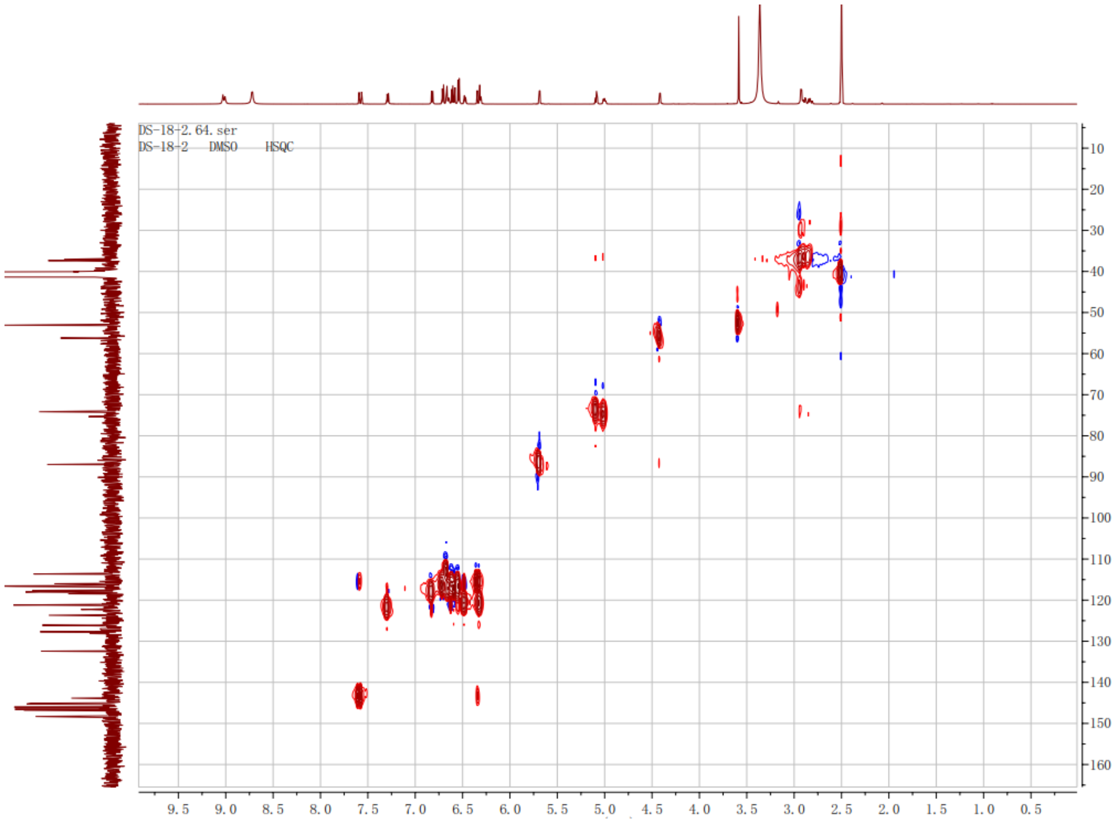

S20. The HMBC spectrum of **4** (DMSO-d_6)

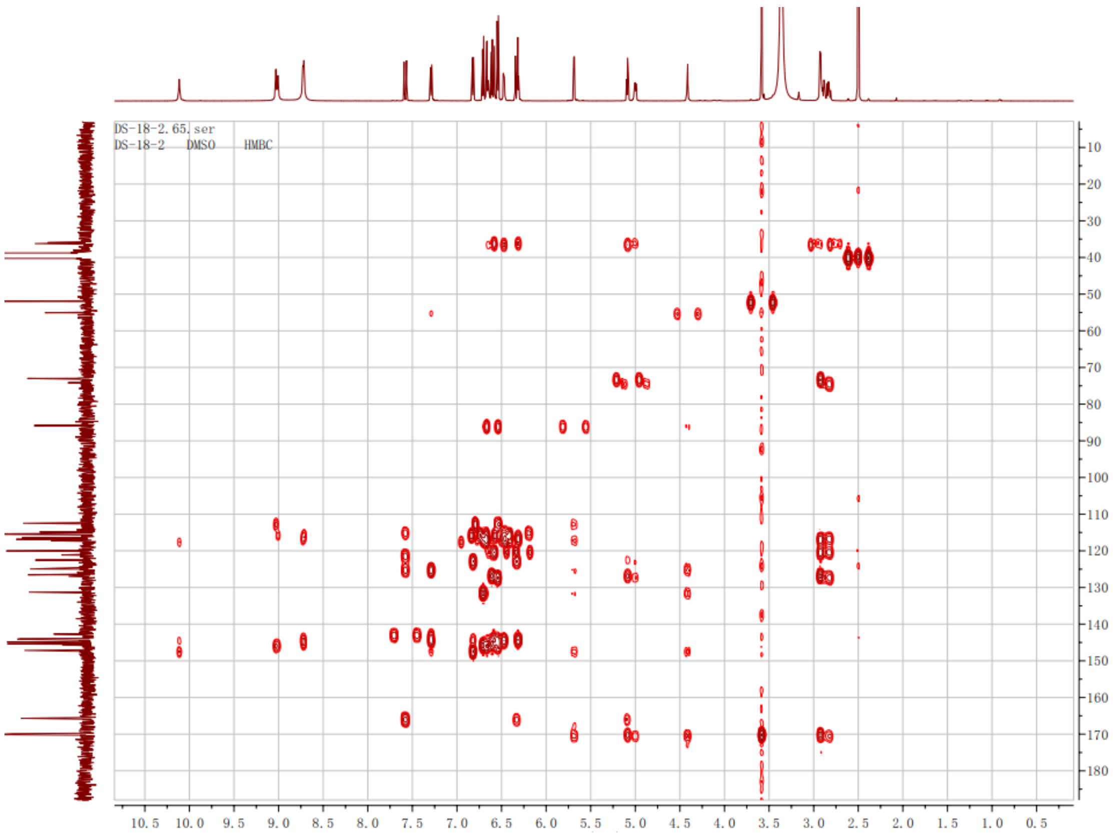

S21. The HR-ESI-MS spectrum of **4**

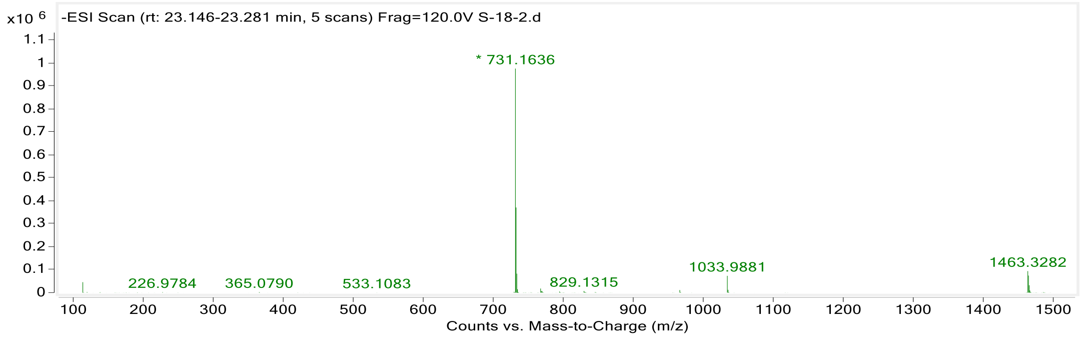

Supplement: Supplementary file 1 [file molecules-23-03274-s001.pdf]
